# Predicting regional somatic mutation rates using DNA motifs

**DOI:** 10.1371/journal.pcbi.1011536

**Published:** 2023-10-02

**Authors:** Cong Liu, Zengmiao Wang, Jun Wang, Chengyu Liu, Mengchi Wang, Vu Ngo, Wei Wang

**Affiliations:** 1 Department of Chemistry and Biochemistry, University of California San Diego, La Jolla, California, United States of America; 2 State Key Laboratory of Remote Sensing Science, Center for Global Change and Public Health, Faculty of Geographical Science, Beijing Normal University, Beijing, China; 3 Bioinformatics and Systems Biology Graduate Program, University of California San Diego, La Jolla, California, United States of America; 4 Department of Cellular and Molecular Medicine, University of California San Diego, La Jolla, California, United States of America; Icahn School of Medicine at Mount Sinai, UNITED STATES

## Abstract

How the locus-specificity of epigenetic modifications is regulated remains an unanswered question. A contributing mechanism is that epigenetic enzymes are recruited to specific loci by DNA binding factors recognizing particular sequence motifs (referred to as epi-motifs). Using these motifs to predict biological outputs depending on local epigenetic state such as somatic mutation rates would confirm their functionality. Here, we used DNA motifs including known TF motifs and epi-motifs as a surrogate of epigenetic signals to predict somatic mutation rates in 13 cancers at an average 23kbp resolution. We implemented an interpretable neural network model, called contextual regression, to successfully learn the universal relationship between mutations and DNA motifs, and uncovered motifs that are most impactful on the regional mutation rates such as TP53 and epi-motifs associated with H3K9me3. Furthermore, we identified genomic regions with significantly higher mutation rates than the expected values in each individual tumor and demonstrated that such cancer-related regions can accurately predict cancer types. Interestingly, we found that the same mutation signatures often have different contributions to cancer-related and cancer-independent regions, and we also identified the motifs with the most contribution to each mutation signature.

## Author summary

Locus-specific epigenetic modifications play critical roles in various biological processes. However, it remains elusive how proteins and their binding motifs regulate such locus-specific epigenetic patterns. A contributing mechanism is that epigenetic enzymes are recruited to specific loci by DNA binding factors recognizing particular sequence motifs (referred to as epi-motifs). Using these motifs to predict biological outputs depending on local epigenetic state such as somatic mutation rates would confirm their functionality. Here, we developed an interpretable neural network model using contextual regression (CR) to predict somatic mutation rates at kilobase resolution using DNA motifs in 13 diverse cancers and identified the most informative motifs particularly epi-motifs. Furthermore, we showed that the genomic regions with significantly higher mutation rates than the predicted values can be used for cancer classification, thus facilitating discovery of the underlying mechanisms. Importantly, this study provides candidate motifs and TFs for the investigation of new mechanisms and the trained CR model is readily applicable to new cancers and identifying cancer-related regions. The CR model can also be applied to other biological questions, such as predicting histone modification using DNA sequences.

## Introduction

Locus-specific epigenetic modifications, such as DNA methylation and histone modifications, play critical roles in various biological processes [1]. While epigenetic patterns are influenced by multiple factors, including nucleosome positioning [2,3], modifying enzymes [3], transcription factors (TFs) [4], non-coding RNAs (ncRNAs) [5], signaling molecules [6] and three-dimensional genomic organization [7,8], the epigenetic modifying enzymes generally do not recognize specific DNA sequence or do not bind to DNA at all and they need to be recruited to specific loci by DNA binding proteins or ncRNAs. Pioneer transcription factors are examples of such proteins that initiate chromatin remodeling and activate regulatory elements in particular loci [9–12]. However, proteins and their binding motifs responsible for establishing or maintaining other types of locus-specific epigenetic patterns largely remain elusive.

Accumulating evidence suggest the importance of DNA sequence features in shaping epigenetic patterns [4,13–20]. DNA motifs associated with epigenetic modifications (referred to as **epi-motifs**) have been documented [21–23]. The readout of epi-motifs is dynamic and dependent upon cellular conditions (e.g. activity of the DNA binding regulator and its access to DNA), and thus is the epigenome. This mechanism is similar to how TFs function: while the TF motifs remain the same, the transcriptional regulation is tissue-specific and dynamic. Successful prediction of gene expression using TF motifs supports the functionality of TF motifs [24,25]. Utilizing epi-motifs to predict biological outputs depending on local epigenetic state, such as somatic mutation rates, would thus help to illustrate their importance in regulating epigenetic locus-specificity.

Somatic mutations are tightly associated with disease phenotypes and are resulted from the interplay between mutagenic processes and DNA repair mechanisms [26–32]. The regional mutation rates are related to various factors, including replication timing, transcriptional activity, nucleosome positioning, chromatin accessibility, histone modifications and protein binding [26–32]. Analysis of mutation rates has been performed at multiple scales. At the megabase scale, high mutation rate is correlated with later replication timing, closed chromatin, strong repressive (e.g. H3K9me3) and weak active (H3K4me1/2) histone marks [33–38]. At the gene scale, reduced mutation rate is associated with high transcription and high H3K36me3 levels [39–42]. At the scale of tens to hundreds of bases, nucleosome positioning is correlated with periodicity of mutation rates [43–49]; furthermore, while high mutation rates are observed at the binding sites of CTCF [40,50,51], ETS family and numerous other transcription factors [52–55], simultaneous analysis of DNA damage and repair suggests that the impact of protein binding varies from no effect to inhibition or stimulation on DNA damage depending on TF and DNA damaging agent [56]. At the smallest scale of several base pairs, previous analyses have uncovered sequence context of somatic mutations and mutational signatures [57–61].

These observations support that chromatin state is tightly correlated with regional mutation rate [29–31]. Such a relationship can be quantified at the megabase scale by machine learning models [34,37,62]. However, at finer scales (e.g. tens of bases to kilobase), no strong correlation between individual epigenetic signals and mutation rates has been observed [34,37,62] and a quantitative model to explain this relationship has not been established. This knowledge gap hinders the understanding of the epigenetic mechanisms that regulate somatic mutation. As protein binding has been suggested to influence the balance between DNA damage and DNA repair rates around their binding sites [52–56], considering DNA motifs recognized by DNA binding proteins may aid in establishing a prediction model for mutation rates. However, it has to overcome the challenge that proteins can have divergent effects on mutation rate upon binding [56].

Given that driver mutations in cancers and other diseases only account for a small portion of all somatic mutations, we hypothesize that (1) the majority of somatic mutations are correlated with the regional features, such as epigenetic state and TF motifs, and the regional mutation rates can be predicted by these relevant features; in other words, somatic mutations in these regions are disease-independent and their occurrence is only related to the local environment rather than the disease state. (2) a relatively small portion of genomic regions contain disease-related mutations and the mutation rates in these regions significantly deviate from the expected values; in other words, these mutations are driven by the disease state and have higher mutation loads than expected from the local environment.

We present here an interpretable deep neural network model that predicts somatic mutation rate at the kilobase scale using DNA motifs. We calculate the mutation rate in genomic regions with distinct epigenetic states as annotated by ChromHMM [3] based on the following rationale. As the epi-motifs are associated with the regional epigenetic state [21–23] and the regional epigenetic state has been shown to be associated with somatic mutation rates [33–38], it is reasonable to use epi-motifs together with other motifs to predict regional mutation rates in the same epigenetic state. If randomly segmenting the genome, a single region likely contains segments in different epigenetic states (e.g. the active enhancers are often 200-300bp long which is only part of a kilobase-long region) and it is thus inappropriate to predict the mutation rate of the entire region using epi-motifs together with other motifs. If randomly segmenting the genome at a high resolution such as 200bp to avoid this problem, the regions are too short to get stable mutation rates and we are not aware of any study that can predict mutation rates at even kilobase resolution using only sequences.

**Fig 1:**
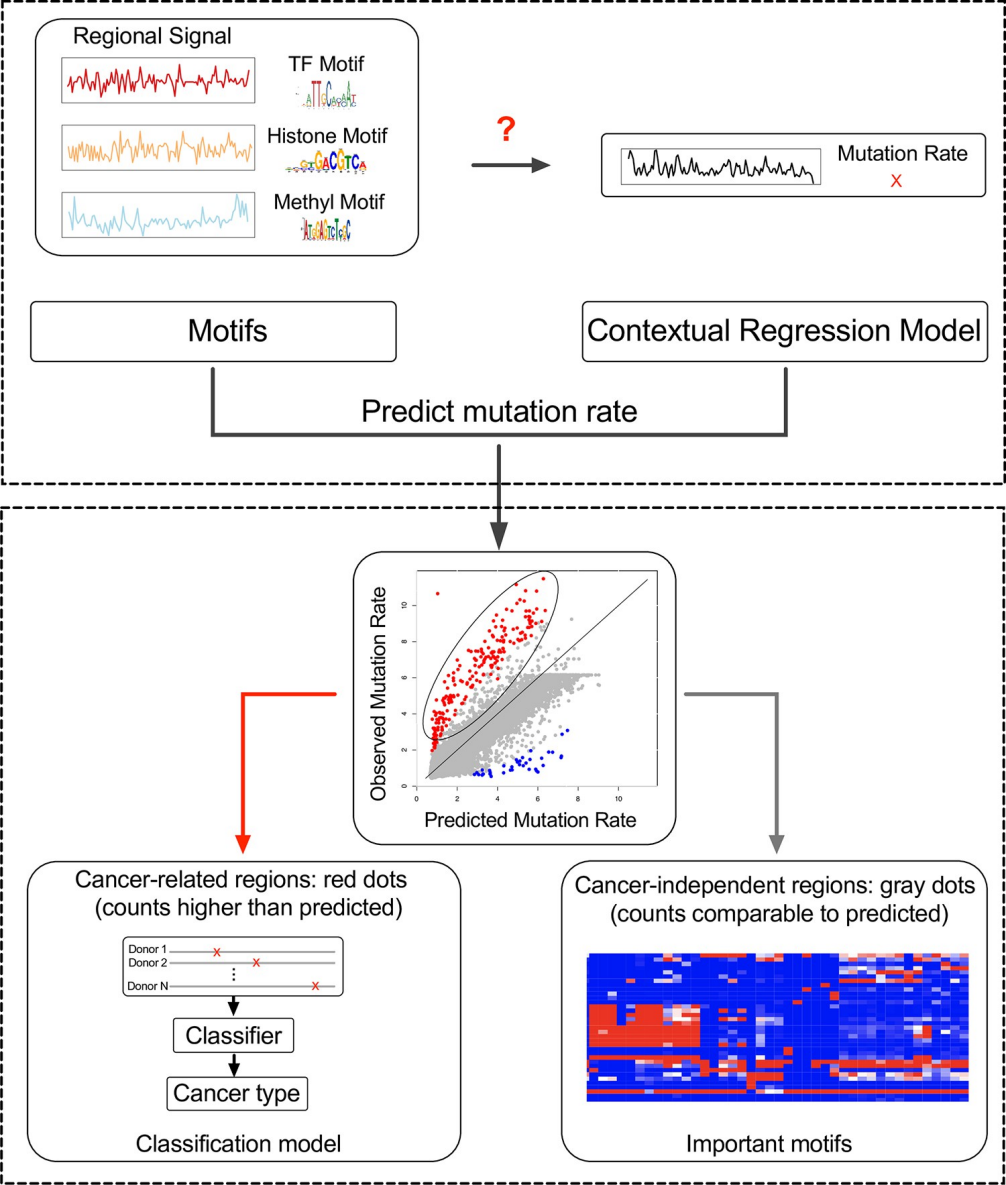
The flowchart of the analysis. Using DNA motifs, including known TF motifs (TF motifs), histone associated motifs (Histone motifs) and DNA methylation associated motifs (Methyl motifs) to represent epigenetic states, we built a contextual regression (CR) model to predict regional mutation rates. As the majority of the mutations are related to the local epigenetic state and independent from the disease state (grey dots), this CR model can quantify the relationship between DNA motifs and somatic mutation rates. Importantly, the CR model revealed the motifs most predictive of somatic mutations (right branch) and the predicted mutation values allowed classification of cancer types using the cancer-related regions with significantly higher mutation rates than predicted (left branch). In the scatter plot, each point represents a training/testing instance, which is the predicted/measured mutation rate of a genomic region. The mutation rate is the log2(MutationRate+1), which is consistent with Fig 2C. The rows of the heatmap are important motifs and the columns are different types of cancers.

We chose DNA motifs as input features because protein binding has been shown associated with the regional mutation rate [29–31]. By building such a predictive model, we aimed to uncover the DNA motifs that enhance or repress somatic mutations (Fig 1). Similar to how the identification of TF motifs important for gene regulation in promoters and enhancers has contributed to our understanding of transcriptional regulation [63,64], we propose that identifying mutation-associated motifs, particularly epi-motifs, will help pave the way towards revealing the molecular mechanisms influencing the rate of regional somatic mechanisms.

Our model has several unique features. First, we not only include known motifs documented in the literature but also de novo motifs associated with DNA methylation and histone modifications (referred to as **epi-motifs**) [21–23]. The inclusion of epi-motifs can approximate the epigenomic signals and would reveal DNA motifs involved in regulating locus-specific epigenetic modifications. Second, our model is interpretable, utilizing a method called contextual regression [65,66]. It can assess the contribution of each motif to the prediction accuracy and determine whether the presence of a motif is associated with an increase or decrease in the regional mutation rate. Third, this model can identify disease-related regions that exhibit exceeding loads of mutations. These regions can be used for classifying disease type.

The objective of this study was threefold: (1) to develop an interpretable deep neural network model that predicts somatic mutation rate at the kilobase scale using DNA motifs, (2) to identify the motifs that are most impactful on the regional mutation rates and (3) to classify cancer types using disease-related regions that contain significant higher mutation rates than predicted values.

## Results

### Regional somatic mutation rates could be predicted using DNA motifs at kilobase resolution

We collected somatic mutations from 1,125 donors, detected through whole-genome sequencing (WGS) from the Pan-Cancer Analysis of Whole Genomes (PCAWG) project [67] (see Online Methods for selection criteria and S1 Table). These donors were associated with 13 tumor types and contained a total of 8,086,632 somatic mutations. To define the genomic segmentation and epigenetic states in the normal tissues corresponding to each cancer type, we took the genomic segmentation defined by ENCODE using ChromHMM [68]. The rationale behind this approach is that the majority of the mutations in cancers are random, and we reasoned that they depend primarily on the local epigenetic state, which can be approximated using normal cells. We observed that, on average, 80.3% of the genome shares similar ChromHMM states between the cancer cell line and their corresponding normal cell line (S2 Table). Because the ChromHMM segments vary in length reflecting the distinct scales of different chromatin states, we calculated and normalized mutation rate in each region as $D_{i}=\frac{C_{i}}{\frac{l_{i}}{1000}\times \frac{T}{10^{6}}}$, where *T* is the total number of somatic mutations in the tumor dataset under consideration, *C*_*i*_ the number of somatic mutations in region *i*, *l*_*i*_ is the length (bp) of this region. It is worth noting that the size of the genomic region is much smaller, with an average 22.7kb (S3 Table), compared to the larger 1Mbp regions used in the previous studies [34,37,62]. This finer scale is expected to better capture the regional epigenetic state and mutation rate.

To build our prediction model, we utilized a comprehensive set of DNA motifs as input features. This set included 1,663 known human motifs from the literature, as well as 310 motifs associated with DNA methylation and 348 motifs associated with histone modifications, as previously identified [22,23] (Online Methods and S4 Table). We trained and tested the contextual regression (CR) model on 80% of all the donors to predict the regional mutation rates, while the remaining 20% of donors were left out for evaluating the model’s capability for classifying cancer types. The framework of this study is shown in S1 Fig.

Contextual regression (CR) is a framework to interpret machine learning models [65,66,69]. It can quantify contributions of features by learning an embedding function that maps each feature vector to a local linear model capable of predicting the target value. The values assigned to each element in the feature vector are considered as context weight and the embedding serves as the classifier of the context. By analyzing the statistics of the context weight, the contribution of each feature can be inferred. CR has been successfully applied to identify important features from neural network models such as those predictive of open chromatin [65] and circular RNA biogenesis [66].

**Fig 2:**
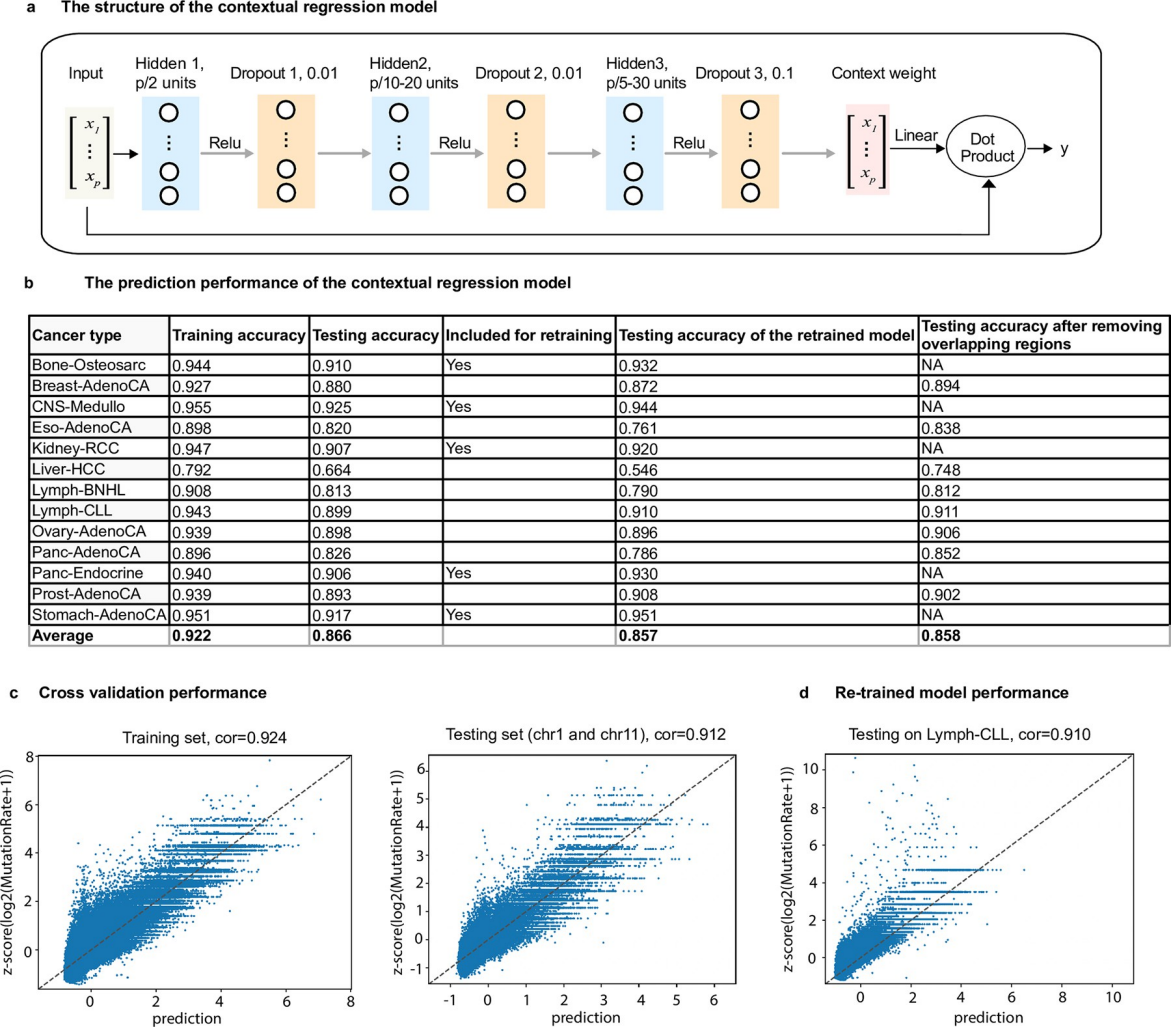
The contextual regression model successfully predicted somatic mutation rates in 13 tumors. (**a**) The structure of the contextual regression model; (**b**) For each tumor type, 10-fold cross validation was performed and the Pearson correlation coefficient was calculated between the predicted and measured values. "Training accuracy" and "Testing accuracy" represent the average of Pearson correlation coefficients in the training, testing datasets respectively. "Included for re-training" indicates which data set was included for re-training the CR model after removing the cancer-related regions (i.e. the regions with mutation rates significantly deviating from the predicted values). "Testing accuracy of the re-trained model" represents the correlation using the re-trained CR model obtained from the interactive procedure (see Online Methods). Because the regions from a tumor type in the test set may overlap with the regions included in the merged dataset for training, the overlapped regions were removed and Pearson correlation coefficients are shown as "Testing accuracy after removing overlapping regions"; (**c**) The scatter plot for one fold from the 10-fold cross validations in which chr1 and chr11 were left out as the testing set; (**d**) The scatter plot for prediction in Lymph-CLL testing set using the re-trained CR model.

Here, we constructed a CR model with a fully connected neural network architecture (Fig 2A). The model consisted of one input layer and 7 hidden layers. The 1^st^, 3^rd^, and 5^th^ hidden layers contained *p*/2, *p*/10-20, *p*/5-30 nodes, respectively (*p* is the number of features). Each hidden layer was activated using the rectified linear unit (ReLU) activation function, and each was followed by a dropout layer (2^nd^, 4^th^, 6^th^ hidden layer) with a rate of 0.01, 0.01 and 0.1, respectively. Dropout layer is a common technique used in model training to prevent overfitting. Dropout rate refers to the percentage of the neurons that are randomly dropped out in the hidden layer. Higher dropout rates mean that more neurons are deactivated. The 7^th^ layer is the Context Weight layer with *p* nodes and a linear activation function. The output was generated by taking the dot product between the input and Context Weight layers (Fig 2A).

We hypothesized that (1) the majority of the somatic mutations are random and independent of cancer (the associated regions are referred to as **cancer-independent regions**), and (2) such random mutations are associated with the local epigenetic state and can be predicted by DNA motifs. To test this hypothesis, we trained the model to predict mutation rates in all regions. We anticipated that an accurate prediction would confirm the dominant majority of regions to be cancer-independent. We then identified **cancer-related regions** as those whose observed mutation rates significantly deviate from the predicted values and the prediction model could be further improved by removing the identified cancer-related regions (see S2 Fig).

To implement this strategy, we first performed the CR prediction with 10-fold cross validations for each cancer type and calculated the Pearson correlation between the predicted and observed mutation rates (see S3 Table). The average Pearson correlation was high, measuring 0.866 (Fig 2B), indicating a dominant majority of cancer-independent regions. To build a universal model for all cancers, we selected 5 cancers with large sample size (donors and regions) and superior prediction performance (average Pearson correlation coefficients > 0.90 in the test sets in the 10-fold cross-validations). These cancers included Bone-Osteosarc, CNS-Medullo, Kidney-RCC, Panc-Endocrine and Stomach-AdenoCA. We merged the regions from these five cancers. To avoid the similar regions present in both training and test sets, we held out 2 or 3 chromosomes for testing while training the model on the other chromosomes. We performed 10 such cross-validations, resulting in average Pearson correlations of 0.926 on the training sets and 0.907 on the test sets, respectively (see S5 Table and Fig 2C). It is worth noting that the ChromHMM regions differ across the 13 cancers. Few exact ChromHMM segments (with exactly the same starting and ending locations in the genome) were shared across all 13 cancers, with 80% of the ChromHMM states unique to only one cancer and a negligible portion (much smaller than 0.01%) occurring in all 13 cancers. Therefore, the features on the regions are distinct for different cancers and their somatic mutation rates can be predicted using the same model.

**Fig 3:**
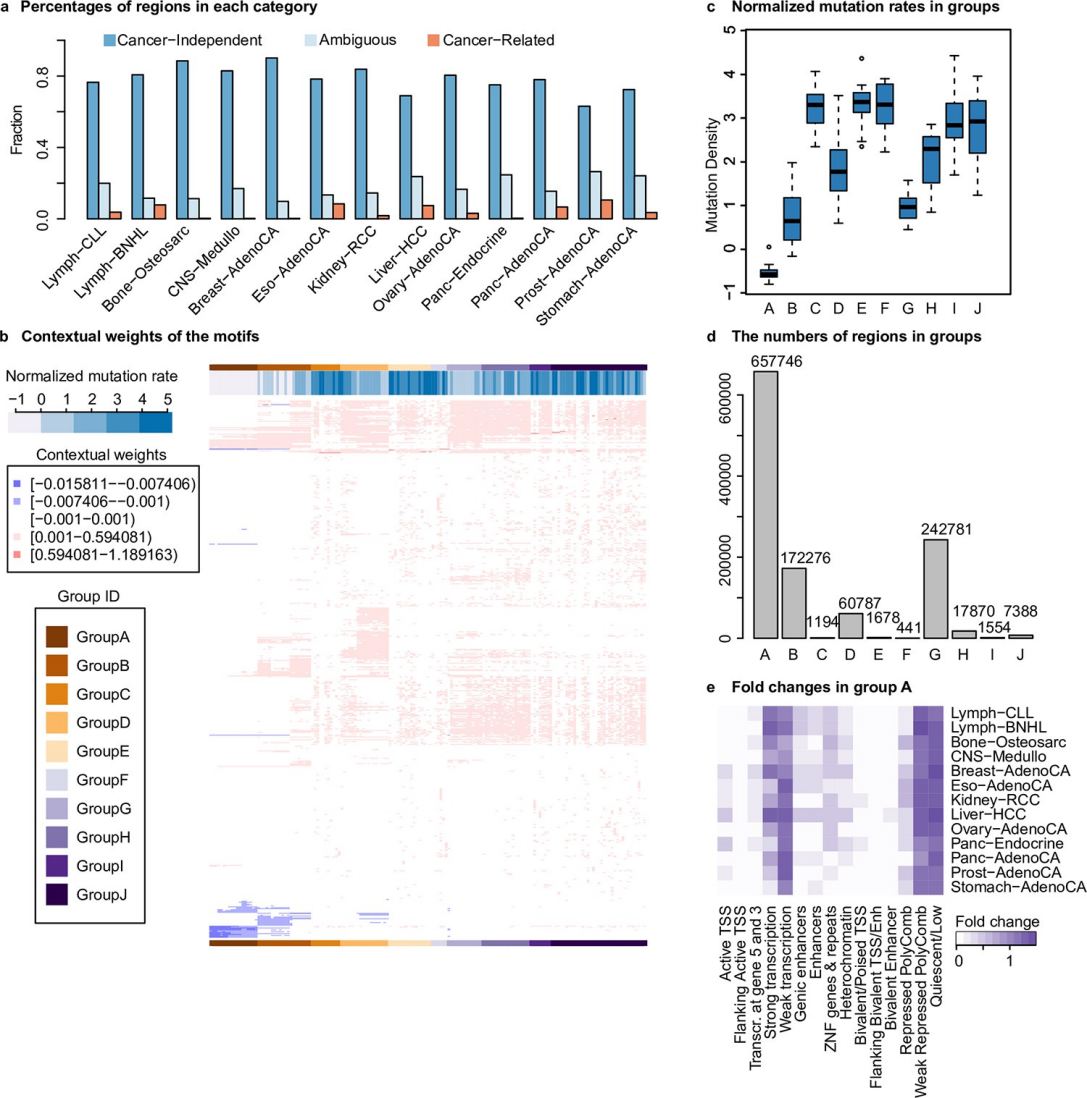
Analysis of the cancer-independent regions. (**a**) The percentage of cancer-independent regions in the 13 cancer types. Percentage is calculated as the number of cancer-independent/related or ambiguous regions divided by the total number of regions in a cancer; (**b**) Cancer-independent regions clustered using the contextual weights of the motifs. For each of the 13 cancer types, the identified cancer-independent regions were clustered into 10 clusters using the Manhattan distance between the feature contextual weight vectors as the similarity metric. Each row is a motif with non-zero contextual weight, each column a cluster, and each entry is the average of a motif’s contextual weights in all the regions in a cluster. The clusters were further clustered into 10 groups; (**c**) The normalized mutation rate of each group, which is the z-score of mutation density (see methods for more details), varies significantly from the lowest in group A to the highest in group G; (**d**) The numbers of regions in the 10 groups; (**e**) The fold change of ChromHMM states in group A and each tumor. The fold change for each ChromHMM state is defined as the percentage of the state in group A divided by the percentage of the state in all the regions in a specific cancer.

Our next focus was on improving the prediction performance for cancer-independent regions. To achieve this, we first identified and removed regions whose observed mutation rates significantly deviated from the predicted values (Online Methods). Subsequently, we re-trained the CR model using the remaining regions (i.e. cancer-independent regions) that better captured the relationship between epigenetic state and random somatic mutations (S2 Fig). We next refined the identification of cancer-independent regions and cancer-related regions using the re-trained CR model. Consistent with our hypothesis, the majority of regions are cancer-independent, with the highest proportion being 90.1% in Breast-AdenoCA and the lowest being 63.1% in Prost-AdenoCA (Fig 3A, S6 Table).

To evaluate the prediction performance using an independent data set, we applied the re-trained CR model to the remaining 8 cancer types (Figs 2B, 2D and S3 and S3 Table,) and achieved an average Pearson correlation coefficient of 0.857. Considering regions included in the 5 training datasets might also appear in the other datasets, we removed all the overlapping regions in the test set, and the average Pearson correlation was not affected and remained high at 0.858 (Fig 2B). Taken together, the CR model successfully captured the relationship between cancer-independent somatic mutation rates and DNA motifs in diverse tissues.

Notably, the prediction performance using DNA motifs alone is comparable to that using Chromatin immunoprecipitation followed by sequencing (ChIP-seq) data of TFs and histone modifications. For example, using 165 TF and histone ChIP-seq of data from GM12878 (downloaded from https://www.encodeproject.org/) as the input features to predict the somatic mutation rates of Lymph-CLL (with GM12878 as the corresponding normal cell for Lymph-CLL), the Pearson correlations on the training and testing datasets were 0.903 (compared to 0.943 using motifs) and 0.871 (compared to 0.899 using motifs), respectively. The slightly lower correlation using ChIP-seq data may be attributed to the smaller number of TFs measured by ChIP-seq compared to the number of available motifs. Since most cancers lack extensive ChIP-seq data in the corresponding normal tissues or cell lines, this observation indicates that using TF and epi-motifs can be useful for approximating the regional epigenetic states in predicting mutation rates.

The analysis of the prediction results indicates that our model does not solely predict average mutation rates in different ChromHMM states. We demonstrated this by showing that the same ChromHMM state exhibits a wide range of mutation rates (S4 Fig). This observation holds true for all the analyzed cancers, not just breast cancer. Additionally, we observed a high correlation between the predicted and measured mutation rates in all regions within the same ChromHMM states (breast cancer as an example in the S5 Fig and each panel is one ChromHMM state). These observations clearly showed that our model can indeed predict mutation rates for individual regions and not just distinguish the mean mutation rates between different ChromHMM states.

Furthermore, we merged the similar motifs to remove redundancy and the model’s performance remained comparable with the original model (S7 Table). We chose to use the non-merged motif set in this study because different versions of the same motifs may represent collaborations of the same TF with different partners.

### Contextual regression identified important features in cancer-independent regions

To identify the most important features in each cancer-independent region, we selected motifs with both the largest contributions to the predicted value and the largest contextual weights: (1) we first selected the top 10% of features with the largest |*β*_*i*_*X*_*i*_|, where *β*_*i*_ is the contextual weight for feature *X*_*i*_. |*β*_*i*_*X*_*i*_| represents the contribution of feature *X*_*i*_ to the predicted mutation rate of this region. (2) Among the top 10% of features with the largest |*β*_*i*_*X*_*i*_|, we selected the top 10% of features with the largest |*β*_*i*_|. In the following analyses, we only focused on the selected motifs and each region was thus represented by a vector composed of the contextual weight value *β*_*i*_ for the selected motifs and 0 for the unselected motifs.

Using the contextual weight profile obtained for each cancer, we performed clustering of the cancer-independent regions using K-means. The value of *k*, the number of clusters, was determined as the elbow point on the elbow curve for each individual cancer. After removing the clusters with small size (less than 10 regions), we obtained a total 163 clusters across 13 cancers. These clusters were further grouped into 10 distinct groups using hierarchical clustering. Group A had the most clusters (18), while group F had the least (6) (Fig 3B, see S6 Fig for the full heatmap). The 10 groups show distinct characteristics. First, the mutation rate is the lowest in group A and the highest in group E (Fig 3C). Second, the number of regions in each group varied significantly, with group A containing the largest number of regions (657,746) and group F having the smallest number (441) (Fig 3D). Third, two ChromHMM states, namely the Weak Repressed PolyComb and Quiescent/Low states, were only enriched in group A across all 13 cancers but not in any other groups (Figs 3E and S7).

**Fig 4:**
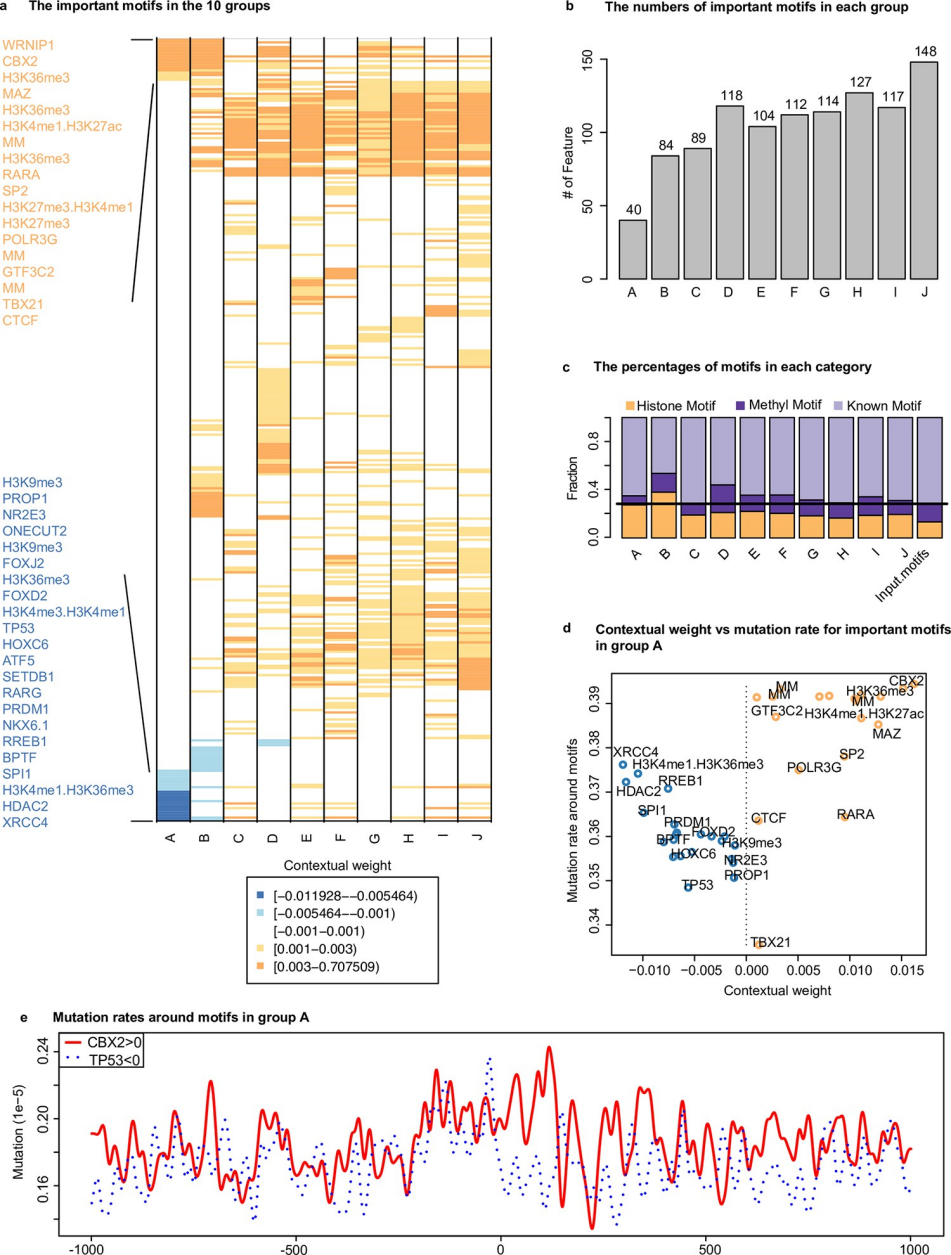
Identification of important motifs in cancer-independent regions. (**a**) The important features in the 10 groups. Blue and orange represent the negative and positive contextual weight, respectively; (**b**) The number of important motifs in each group; (**c**) The percentages of motif categories in each group; (**d**) The average mutation rates around the motifs with positive contextual weights are higher than those around the motifs with negative contextual weights in the group A regions; (**e**) Mutation rates around the motif sites (1kbp at each side of the motif) in the group A regions. The red and blue lines represent the motifs with positive and negative contextual weights, respectively. The motif site is at the center.

To identify the important features in each group, we calculated the average of contextual weights across clusters for each group (Fig 4A and S8 Table). This analysis led to the identification of 336 unique motifs distributed across the 10 groups, with group J having the highest number of motifs (130) and group A having the smallest number (41) (Fig 4B). Interestingly, although epi-motifs (motifs associated with histone modification and DNA methylation) accounted for only 28% of the input motifs, their percentage increased significantly to average 35% (one-proportion z-test *p*-value = 5.1×10^−7^) among the important motifs in the 10 groups. This increase was particularly prominent in group A, B, F and I (Fig 4C). This finding supports the notion that epi-motifs play a crucial role in establishing regional epigenetic states [22,23].

To gain deeper insights, we focused on group A due to its largest number of regions. In this group, we identified 18 motifs with positive contextual weights and 22 motifs with negative contextual weights (Fig 4A). The sign of the contextual weights indicates positive or negative association between the input feature and the predictive value. Therefore, our analysis suggested that the 18 and 22 motifs would have opposite impacts on somatic mutations. We then examined the mutation rates around these motifs and found that motifs with positive weights were associated with significantly higher mutation rates compared to those with negative weights (*p*-value of 4.5×410^−6^ from Student’s t-test) (Fig 4D). Furthermore, we performed pairwise comparison between all the possible positive- and negative-weighted motif pairs. In 62.6% of all the pairs (248/396, using a *p*-value cutoff of 0.05 from Student’s t-test), the mutation rates around the motif sites (upstream 50bp to downstream 50bp) for positive-weighted motifs were higher than the paired negative motifs. One motif pair of CBX2 (positive coefficient) and TP53 (negative coefficient) is shown as an example in Fig 4E. The mutation rate is significantly higher (*p*-value of 1.51×10^−4^ from Student’s t-test) around the CBX2 motifs than that around TP53 (i.e. M6403_1.02) motifs in group A. TP53 is known to play a significant role in repairing damaged DNA [70], supporting the reasonable negative association between TP53 motifs and mutation rate.

Among the important motifs in group A, we observed 6 positive- and 5 negative-weighted histone-motifs (Fig 4A). The positive association of the H3K27me3 motifs and the negative association of H3K4me3.H3K4me1 and H3K4me1.H3K36me3 motifs with the mutation rate are not surprising and consistent with the literature. The positive association of 1 H3K4me1.H3K27ac and 3 H3K36me3 as well as negative association of 2 H3K9me3 motifs suggest the relationship between histone modification and somatic mutation at the kilobase scale is more complex than expected from the previous megabase-scale studies. Interestingly, we also found 3 DNA methylation motifs (MM) positively associated with somatic mutation rates, indicating the possible roles of DNA methylation on affecting DNA damage and repair.

### The CR model allows identification of cancer-related genomic regions and motifs

**Fig 5:**
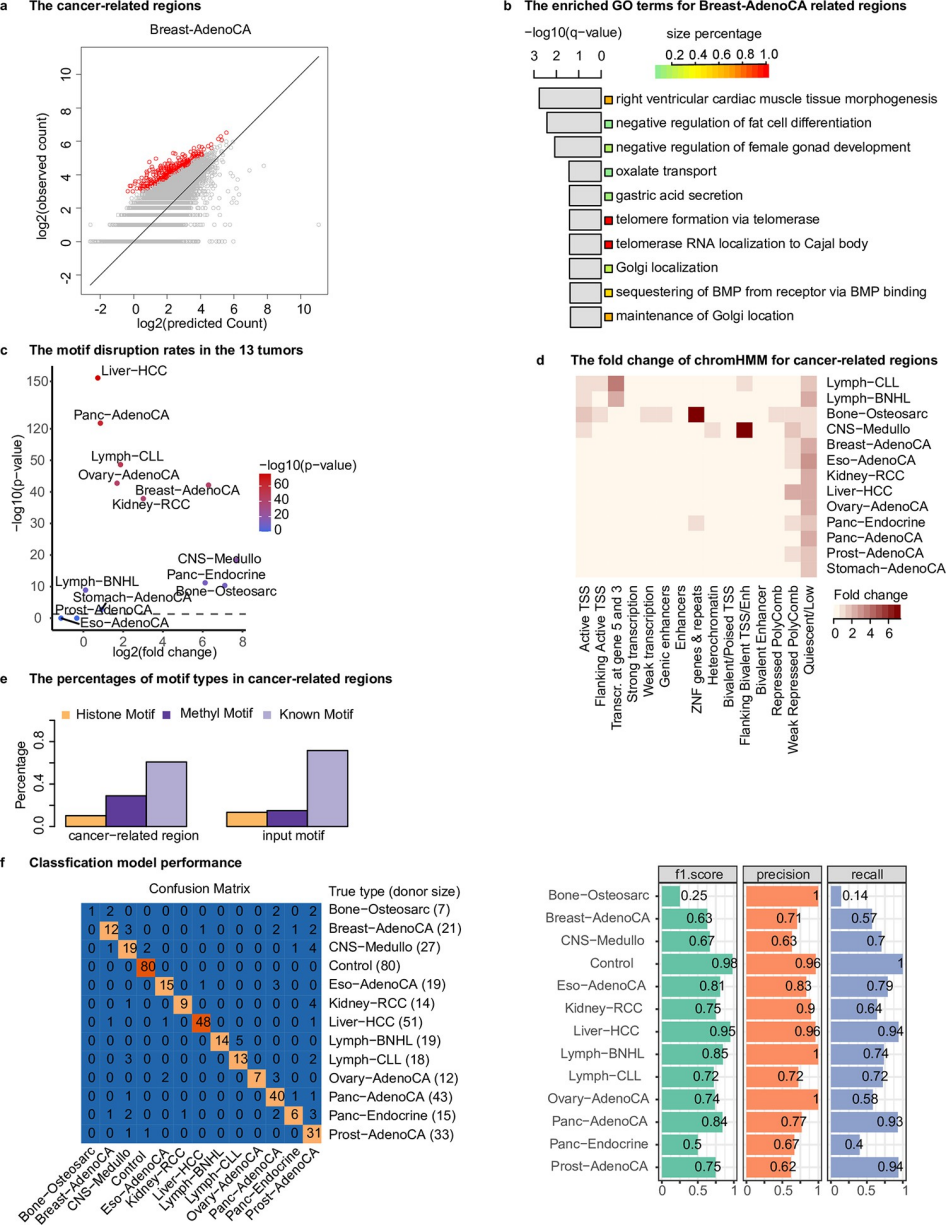
Analysis of the cancer-related regions. (**a**) The identified cancer-related regions in Breast-AdenoCA (red dots); (**b**)The enriched pathways for the cancer-related regions in Breast-AdenoCA; (**c**) The fold change and *p*-value for the motif disruption rates in the 13 tumors. The red line represents the *p*-value of 0.05; (**d**) The fold change of chromHMM state (same as that in Fig 3E) for the cancer-related regions in each tumor; (**e**) The percentages of motif types that were significantly disrupted in cancer-related regions; (f) The classification model performance. The confusion matrix for the classification model using the 150 selected cancer-related regions on the testing dataset. Rows and columns correspond to the true and predicted tumor types, respectively. Values are the number of donors classified correctly. For example, for the Prost−AdenoCA, 31 donors were correctly classified.

The analysis of the predicted mutation rates allowed us to identify cancer-related regions in each tumor type (see Online Methods). The percentage of these regions varied across cancer types, ranging from 0.2% in CNS-Medullo to 10.5% in Prost-AdenoCA, with an average of 4.1% (Fig 3A). The small proportion of these cancer-related regions confirms our hypothesis that the majority of genomic regions are cancer-independent. One example for cancer-related regions in Breast-AdenoCA is shown in Fig 5A. We analyzed the biological processes in cancer-related regions for each cancer type (S9 Table) and the enriched ones for Breast-AdenoCA are shown in Fig 5B, including those relevant to breast tissue and/or breast cancer such as "right ventricular cardiac muscle tissue morphogenesis", "negative regulation of fat cell differentiation", and "negative regulation of female gonad development" as well as "telomere formation via telomerase" and "telomerase RNA localization to Cajal body". Notably, the key gene WRAP53 is also found in the Breast-AdenoCA-related regions, which is related to the increased risk of breast cancer [71–73].

We also found that the disruption rate of motifs by somatic mutations in cancer-related regions was significantly higher than that in cancer-independent regions for 10 out of 13 tumor types (*p*-value < 0.05, Fig 5C). The disruption rate of motifs for cancer-related regions is defined as the number of all motif binding sites overlapped with somatic mutation divided by the number of all motifs binding sites and the number of somatic mutations (see Online Methods). This observation supports the hypothesis that the DNA motifs play a role in shaping the epigenetic state, and mutations that disrupt these motifs may be associated with tumorigenesis.

To identify the most significantly disrupted motifs in the cancer-related regions for each cancer, we selected motifs that had a disruption rate in the top 5% of all motifs with a *p*-value < 0.05 (*p*-value calculated from the Student’s t-test for the alternative hypothesis of the disruption rate in cancer-related regions larger than that in cancer-independent regions). Among the 342 motifs obtained in 13 cancer types, 60.8% of them were known motifs, a decrease from 72% in the input motifs (Fig 5E). 102 (28.9%) DNA methylation associated motifs represented a significant increase from 13% in the input motifs. Notably, all of them are unmethylated motifs (i.e. UM motifs), which are known to be associated with low DNA methylation level [23]. This observation is consistent with the well-established phenomenon of hypomethylation being frequently observed in both highly and moderately repeated DNA sequences, including heterochromatic DNA repeats, in cancer [74]. The most common motifs found across cancer types included one known motif (E2F1, motif ID: M4536_1.02), two unmethylation motifs (UM_235.9_3.32_0.65_1_SGCWCGCGGCGGC and UM_326.6_2.71_0.59_6_CGCGCCCCGY). E2F1 is known to play a crucial role in cell cycle regulation [75] and DNA repair [76]. Somatic mutations in E2F1 binding sites could potentially result in dysfunction of E2F1. Additionally, several motifs were found to be specifically disrupted in certain cancer types (S10 Table), such as M2321_1.02 (TP63) in Bone-Osteosarc, M6446_1.02 (RARG) in Breast-AdenoCA, and M5371_1.02 (EGR4) in Lymph-CLL.

It is interesting that the cancer-related regions in most cancer types were enriched in the ChromHMM state of "Quiescent/Low", which typically exhibits little or low epigenetic signals. Furthermore, each cancer type also showed its own specific epigenetic state, as reflected by the enrichment of different ChromHMM states. For example, "Transcription at gene 5’ and 3‴ is the most enriched state in Lymph-CLL and Lymph-BNHL, while "ZNF genes & repeats" was enriched in Bone-Osteosarc and Panc-Endocrine (Fig 5D).

### The cancer-related regions are predictive of cancer types

The cancer-related regions are presumably important in cancers considering the cancer-related regions are those having significantly higher mutation rates than the predictive mutation values. Therefore, we examined whether these regions could be predictive of cancer types. Twelve cancers with WGS data with >30 donors each were used for multi-class classification of cancer types, with Stomach-AdenoCA excluded due to the small sample size. Additionally, 400 healthy donors from GTEX were included as controls. Using the mutation counts in the 67,890 cancer-related regions (which collectively spanned the entire genome, S6 Table), a Gradient Boosting Decision Tree model was trained for classifying the 12 tumor types and controls. The training and evaluation of the classification model were performed on 80% of the cancer donors (the dataset used in CR model construction) and controls, while the remaining samples (independent data not used in the CR model construction) were utilized as the testing set. A systematic search of the hyperparameter space (27,000 combinations of 6 parameters, S11 Table and method section) was conducted using 5-fold cross-validation on the training data (i.e. the 80% of the 1,382 cancer donors and 320 control donors). The best parameter combination was selected based on the minimal difference of accuracy between the training and validation datasets to avoid overfitting. The prediction accuracy on the test data in the 5-fold cross-validations was 0.865. We re-trained the classification model using all the 80% data with the best parameter combination and its prediction accuracy on the left-out 20% dataset was 0.858.

The feature selection process was conducted using the feature importance metric of Gradient Boosting Decision Tree, and 150 regions (accounting for 5.8% of the whole genome) were selected for re-training the classification model. We also performed the parameter tunings using 5-fold cross validation on the 80% dataset. The accuracy on the testing samples was 0.813 using the best parameter combination (S12 Table). Subsequently, the classification model was re-trained using the 80% dataset with the best parameter combination and its prediction accuracy on the left-out 20% donors was 0.822 (Fig 5F). The recall for each class on the testing dataset ranged from 0.14 in Bone-Osteosarc to 1.00 in control, with a median of 0.72. The precision ranged from 0.62 in Breast-AdenoCA to 1.00 in Bone-Osteosarc, Lymph-BNHL and Ovary-AdenoCA, with a median of 0.83. The F1 score, a comprehensive metric, ranged from 0.25 in Bone-Osteosarc to 0.98 in Control, with a median of 0.75. It is not surprising that the best performance was achieved on control samples, as distinguishing normal samples from tumor samples is generally easier than differentiating between different types of tumors. The performance on Bone-Osteosarc was the worst, which was likely due to small sample size (only 28 donors in training and 7 in testing). Overall, the performance of the model is satisfactory, especially considering that it only utilized 5.8% of the genome for classification. These results further validate the model’s robustness and demonstrate that the predicted mutation values can serve as a reference for identifying regions with aberrantly high mutation rates, which may be associated with specific cancer types.

## Discussion

Distinct from the previous analyses, we showed for the first time that DNA motifs can be predictive of regional somatic mutation rates at the kilobase scale. The DNA motifs include known TF motifs as well as epi-motifs that are associated with histone modifications or DNA methylation. We showed that the prediction performance is comparable to that using histone and TF ChIP-seq data. Considering that genome sequencing is much easier than ChIP-seq experiments in tumor tissues, our model provides a powerful approach to quantify the relationship between somatic mutations and epigenetic state.

A remarkable aspect of the study is the revelation of the pivotal roles played by epi-motifs in predicting regional somatic mutation rates. Despite accounting for only 28% of the input motifs, epi-motifs constitute an average of 35% in the most predictive motifs across the 10 groups. Moreover, the significantly higher frequency of disruption of DNA methylation associated motifs (28.9%) in the cancer-related regions, compared to their percentage (13%) in the input motifs, suggests their pivotal roles in shaping the regional epigenetic state and deciding the locus-specific modifications. These important epi-motifs identified from this analysis can guide future studies to investigate the proteins that bind to these motifs and recruit epigenetic enzymes to specific loci, thereby initiating local changes in the epigenetic state. The success of this study also suggests that it is possible to use epi-motifs as the surrogate of local epigenetic state for predicting other observable measurements.

We hypothesized that the majority of somatic mutations are random and only depend on the regional epigenetic state in the normal cells/tissues. In other words, the majority of the genomic regions containing somatic mutations are cancer-independent. This hypothesis was supported by the high correlation between the predicted and measured mutation rates across all the regions. This kilobase-scale relationship is general across cancers as indicated by the successful prediction on cancers not included in the training dataset. Such a relationship was not previously uncovered in megabase-scale analyses from previous studies.

Furthermore, the contextual regression model provides a framework to interpret the neural network predictions and identify the most predictive features. Using the contextual weights of the predictive motifs, we were able to cluster the genomic regions that share similar motif contribution profiles on predicting mutation rates. The regions in the same cluster are presumably regulated by similar mechanisms, akin to genes sharing similar expression profiles across cell types. In fact, by analyzing these clusters, we observed that the impact of protein binding on regional mutations can be positive, negative or neutral. While the previous study reported that TF binding would block DNA repair proteins in the open chromatin regions to increase mutation rates [55], we found there exist proteins/motifs whose occurrence in the open chromatin regions is associated with lower mutation rates. This observation highlights the importance of genetic and epigenetic context on impacting regional mutations.

Importantly, the predicted mutation rates from the contextual regression model provide a quantitative background to identify cancer-related regions in a particular cancer. These regions exhibit significantly higher mutation rates than expected from the regional epigenetic states in the corresponding normal tissue. The CR model learns the relationship between motifss and mutation rate. For each region from each cancer, it has its own background mutation rate based on its own features. Therefore, if the observed mutation rate is higher than the background, the region may be related to the mechanism of this cancer and thus called cancer-related region. Because regions with higher mutation rates in cancer tissues compared to the normal tissues can be resulted from various causes and might be irrelevant to cancer, using the predicted mutation rates as the reference would allow distinction between regions within cancer tissues and uncover those directly related to cancer. While investigating the underlying mechanisms in these regions is not the focus of this study, the identified known and epi-motifs can help mechanistic analysis in the future and elucidate region-specific factors regulating somatic mutation rates. Based on these cancer-related regions, we showed that only using 150 regions that account for only 5.8% of the human genome could predict cancer types with a satisfactory performance. This result provides a potential diagnosis tool using targeted sequencing.

Mutational signatures have been widely adopted to characterize the preference of mutation types in cancers. Therefore, we performed mutational signature analysis on 3 types of regions, i.e. cancer-independent regions, cancer-related regions and all the regions. The association of cancer-independent regions with the mutational signatures is more similar to that of all regions than cancer-related regions, which is not unexpected. The relative contribution of mutational signatures can be quite different between these two types of regions within the same cancer type. For example, signature 2 contributes more in cancer-related regions in Bone-Osteosarc, compared to that in cancer-independent regions (S8 Fig). Motifs with significant contributions to each mutational signature are listed in Github (https://github.com/Wang-lab-UCSD/SomaticMutation/tree/main/results/supplementaryTables/13_cancers_motifs_contribution_30_SBS.xlsx). It is obvious that the two approaches (CR model and mutational signatures) provide complementary information and the motifs/their binding proteins uncovered by the CR model can guide mechanistic study of mutational signatures in specific regions.

During the submission of this work, Sherman et al. [77] published a deep learning method to predict mutation rates at 10kbp resolution. Our study is different from Sherman et al. in the following aspects and the two studies are highly complementary. First, our model only uses DNA motifs and epigenomic data of 6 histone marks in the corresponding normal cell types for each cancer type. In contrast, Sherman et al. used much more data, including 723 chromatin marks from 111 tissues to train their model as well as replication timing in 10 cell lines and average nucleotide and CG content in the reference genome. Our model uses much less features but can achieve a slightly better prediction performance (a mean Pearson R^2^ = 0.736, i.e. R = 0.858, Fig 2B and S3 Table in our manuscript compared to a mean Pearson R^2^ = 0.706, i.e. R = 0.84, in Sherman et al.). Second, our model can be applied to new cancer types without retraining and only requires the epigenetic state in the corresponding normal cells as shown in our paper that the model trained on 5 cancer types was successfully applied to predict the remaining 8 cancer types. In contrast, Sherman et al. has to re-train the entire model to include new cancer types. Third, our study focuses on understanding how the DNA motifs, particularly the epi-motifs, contribute to regional somatic mutation rates and the contextual regression model provides an interpretable model that directly derives the important motifs. In contrast, Sherman et al. focused on uncovering driver mutations.

In this study we used ChromHMM to segment the genome and annotate the epigenetic states. It is worth noting that the analysis results should be insensitive to the variability of chromatin states. Our model considers the mutation rates of individual regions and does not require a distinction between different states, such as enhancer subclasses. It might be interesting to compare different segmentation methods and use different numbers of ChromHMM states to repeat our analysis in the future.

Taken together, we developed an interpretable neural network model to successfully predict somatic mutation rates at kilobase resolution using DNA motifs in 13 diverse cancers and identified the most informative motifs particularly epi-motifs. Furthermore, we showed that the genomic regions with significantly higher mutation rates than the predicted values can be used for cancer classification, thus facilitating discovery of the underlying mechanisms. The availability of additional WGS data in cancer samples and epigenomic data in the corresponding normal samples would allow further improvement of the model performance and generality. Furthermore, this study provides candidate motifs and TFs for the investigation of new mechanisms and the trained CR model is readily applicable to new cancers and identifying cancer-related regions. The CR model can also be applied to other biological questions, such as predicting histone modification using DNA sequences. Interestingly, we found that the same mutation signatures often have different contributions to cancer-related and cancer-independent regions, and we also identified the motifs with the most contribution to each mutation signature.

## Materials and methods

### Somatic mutation data

The somatic mutation data of 2,583 donors analyzed by the Pan-Cancer Analysis of Whole Genomes Consortium (PCAWG) were downloaded from the International Cancer Genome Consortium (ICGC) data portal [78] (https://dcc.icgc.org/). This was the largest data set when this analysis started. The tumor types for the donors were retrieved from S1 Table in reference [67]. We filtered the data using the following criteria: (1) donors with metastatic tumors were removed because in this study we focused on the primary tumors; (2) outlier donors with extremely high or low somatic mutation numbers were discarded to avoid bias. An outlier was defined as a data point located outside 1.5 times the interquartile range above the upper quartile and below the lower quartile for a tumor type; (3) tumor types with less than 5 donors or without ChromHMM segmentation in the corresponding normal tissues were not included; (4) only WGS data were kept if a donor had both WES and WGS data; (5) removing tumor types if the number of the total somatic mutations in all the donors of that tumor type was less than 30,000. As a result, 1,125 donors from 13 different tumor types remained for model training and testing (S1 Table). Somatic mutations in the blacklisted regions were removed [79]. While it is true that these 13 cancer types may or may not be fully representative of somatic mutation rates in all tissues, it is important to note that the main objective of this study was not to select cancer types as representatives for different tissues. Instead, our study aimed to develop a predictive model to understand how DNA motifs contribute to regional somatic mutation rates across a diverse set of cancers.

### DNA Motifs

We included 1731 human motifs of DNA binding proteins documented in the CIS-BP database [80] and another 55 motifs from Factorbook [63]. We also added 313 motifs associated with DNA methylation [23] and 361 motifs associated with histone modifications [22] that were identified in our previous studies. These motifs were used to approximate the epigenetic state. We used FIMO [81] to scan these total 2460 motifs against hg19. With a *p*-value cutoff of 10^−5^, 2,321 motifs having at least one occurrence were used for the following analyses (S4 Table).

### Genomic segmentation using ChromHMM

The core 15-state ChromHMM segmentations were downloaded from https://egg2.wustl.edu/roadmap/web_portal/. For kidney and prostate gland, the ChromHMM segmentations were not available from the website. To be consistent, we applied the core 15-state trained ChromHMM model to these 2 tissues, which was downloaded from https://egg2.wustl.edu/roadmap/data/byFileType/chromhmmSegmentations/ChmmModels/coreMarks/jointModel/final/model_15_coreMarks.txt. The data for these 2 tissues were downloaded from the ENCODE portal (S13 Table).

### Somatic mutation density and feature calculation

We calculated the somatic mutation density in a given set of cancer patients as the following. Let *R*_*i*_ denote the segmented region *i*, *i* = 1,…,*N*, *l*_*i*_ the length (bp) of *R*_*i*_, and *C*_*i*_ the number of somatic mutations in all the donors in region *i*. The somatic mutations were downloaded from PCAWG. The regional somatic mutation density *D*_*i*_ was computed as $D_{i}=\frac{C_{i}}{\frac{l_{i}}{1000}\times \frac{T}{10^{6}}}$, where *T* is the total number of somatic mutations in all the donors of this dataset. We added a pseudo-count to *D*_*i*_ and defined $D_{i}^{\left(1\right)}=log2(D_{i}+1)$. We then calculated $Y_{i}=\frac{{(D}_{i}^{\left(1\right)}-Mean)}{STD}$ as the response variable in the model by z-score transformation, where *Mean* is the average of $D_{i}^{\left(1\right)}$ and *STD* is the standard deviation error of $D_{i}^{\left(1\right)}$.

Let *M*_*j*_ be the motif *j*, *j* = 1,..,*p*. $C_{i,j}^{\prime }$ is the median *p*-value of all occurrences of *M*_*j*_ in the region *R*_*i*_ and the *p*-value was calculated from FIMO [81]. We used -log10($C_{i,j}^{\prime }$) in each region as the input features to predict *Y*_*i*_.

### Construction, training and testing of the CR model

The architecture of CR model was shown in Fig 2A. We used Adam as the optimization algorithm for training the CR model. Adam is a popular and efficient optimization algorithm commonly used in neural network training. It is well suited for problems that are large in terms of data/parameters. The choice of Adam was based on its strong performance in similar tasks. To ensure the optimal performance of the CR model, we conducted a grid search of hyperparameters, including the dropout rate. Dropout is a regularization technique used to prevent overfitting in neural networks by randomly setting a fraction of the neurons’ output to zero during training. We tested different dropout rates and evaluated the model’s performance. We selected three cancers (Bone-Osteosarc, Breast-AdenoCA, and Liver-HCC) covering the range of the prediction performance. We tested five different dropout rates and the test set accuracies are very similar, which shows the robustness of the model training to dropout rates. The rationale of Contextual regression (CR) is that CR can quantify feature contributions by learning an embedding function to map each feature vector to a linear model that can predict the target value. The values assigned to each element in the feature vector are considered as context weight and the embedding serves as the classifier of the context. By analyzing the statistics of the context weight, the contribution of each feature can be inferred.

The details of the training and testing of the CR model is shown in S1 Fig. Briefly, we divided the donors randomly into two sets: 80% for model training/testing and the rest for independent testing. Using the 80% donors, we trained and tested CR models for individual cancer types using 10-fold cross validations (Step 1 in S1 Fig). The best performed 5 cancers were selected to train a universal model on all the regions in these 5 cancers (Step 2). As the majority of the regions in the cancers could be accurately predicted using the motifs and the ChromHMM segmentation in the corresponding normal cells, it confirmed our hypothesis that somatic mutations in the majority of the genome are cancer-independent. To better capture the relationship between the somatic mutations and epigenetic state, we removed the regions, i.e. cancer-related regions, whose predicted mutations rates significantly deviated from the observed values (Step 3). We further analyzed important motifs with high CR weights (Step 4). We then trained and tested a classification model to distinguish cancer types using the cancer-related regions on the 20% of the donors that were not used to select these regions as an independent test (Step 5).

### Assessing the CR model performance

In each dataset, we performed cross validation to assess the model performance, in which 10% of the segmented regions were held out for testing. Because there were overlapped ChromHMM regions from different tumor types, we partitioned the samples from these 5 tumor types based on the chromosomes to avoid the overlapped regions presenting in training and testing datasets. Two or three chromosomes were randomly left out for testing while the other chromosomes were used for training CR models. We repeated such cross validation 10 times. The specific partitions of training/testing dataset and performances were listed in S5 Table.

### Identification of cancer-related and cancer-independent regions

To identify the cancer-related and cancer-independent regions, we used an iterative procedure. First, we trained a CR model using all the regions from the merged dataset (the first iteration). As the majority of the regions are cancer-independent regions, using all the regions to train the model would not significantly impact the accuracy. Assuming the cancer-independent somatic mutation counts in a specific region follow a Poisson distribution, we estimated the parameter lambda (i.e. the expectation for Poisson distribution) using the predicted counts that were converted from the predicted mutation rates. Based on this Poisson distribution, we calculated a *p*-value for the observed mutation count. If a region had a *p*-value (upper-tail or lower-tail) < 0.1, it was considered as either cancer-related or ambiguous and thus removed from the training set. We retrained the CR model using the remaining regions that presumably contained more cancer-independent ones in the second iteration. Repeating this procedure would continue improving the model and removing regions that are cancer-related or ambiguous. We found that this procedure converged fast as the average mean squared error (MSE) on the testing dataset reached the plateau at the second iteration, indicating that the model became stable. Therefore, we took this model trained using two iterations as the final model for the following analyses.

We used the predicted mutation rates from the final CR model as the background and re-calculated the *p*-value for each region in each tumor. A region was called cancer-independent if its *p*-value (upper-tail and lower-tail) > 0.1, cancer-related if the FDR from the upper-tail for a region was <0.01. The other regions were ambiguous regions and not included in any further analysis.

### Somatic mutation calling for the GTEX data

We downloaded the GTEX WGS data from dbGap (accession number phs000424.v8.p2). We aligned the sequencing reads to hg19 and called somatic mutations using the GATK best practice workflow [82]. We removed the reads that originated from duplicates of the same DNA fragments using MarkDuplicatesSpark function in GATK. Base (Quality Score) Recalibration was conducted for correcting any systematic bias observed in the base quality scores. We followed the guideline of how to call somatic mutations using GATK4 Mutect2 (https://gatk.broadinstitute.org/hc/en-us/articles/360035889791—How-to-Call-somatic-mutations-using-GATK4-Mutect2-Deprecated-). We called candidate variants using Mutect2, which is designed specifically for somatic mutation calling by the GATK group. FilterMutectCalls was then applied to identify variants from artifacts, such as those resulting from alignment, strand and orientation bias, polymerase slippage, and germline variants. The tool uses the annotations within the callset and applies preset thresholds that are tuned for human somatic analyses. This generated a VCF file with a FILTER field. The true positives were labeled with PASS in the FILTER field. Funcotator was used to add annotation to these variants, such as dbSNP and gencode. Lastly, we only considered somatic mutations with a FILTER flag PASS and obtained somatic mutations for 400 donors.

### Disruption rate of the motifs

In the cancer-related regions for a given patient, the disruption rate of a motif was calculated as C/(M*N), where C is the number of motif binding sites overlapped with somatic mutations in the cancer-related regions in this patient, M is the total number of motif binding sites and N is the total number of somatic mutations in the cancer-related regions for this patient. Similarly, we calculated the disruption rate in the cancer-independent regions. To test whether the disruption rate in the cancer-related regions was higher than in the cancer-independent regions, paired-T test was used to compute the *p*-value for each tumor type with the patients as samples. This way, we identified significantly disrupted motifs for each cancer type.

To evaluate whether all motifs were significantly disrupted in one type of cancer, we performed the above analysis for all the motifs. Specifically, given the cancer type and a patient, the disruption rate for all motifs was defined as C/(M*N), where C is the number of all motif binding sites overlapped with somatic mutations in the cancer-related regions in this patient, M is the total number of all motif binding sites and N is the total number of somatic mutations in the cancer-related regions of this patient. The disruption rate in cancer-related regions is calculated in the same way. Paired-T test was used to evaluate the significance for each tumor type with the patients as samples. The *p*-value cutoff was set to 0.05.

### Gradient boosting decision tree

A gradient boosting decision tree was trained to classify cancer types using the scikit-learn package [83]. There were six parameters in the model, including (1) learning rate (denoted as learning_rate); (2) the minimum number of samples (or observations) required in a node to be considered for splitting (min_samples_split); (3) the minimum samples (or observations) required in a terminal node or leaf (min_samples_leaf); (4) the maximum depth of a tree (max_depth); (5) the fraction of observations to be selected for each tree (subsample); (6) the number of sequential trees to be modeled (n_estimators).

We selected the optimal values of the parameters with the best classification performance: when using all the cancer-related regions as features: learning_rate = 0.012; min_samples_split = 150; min_samples_leaf = 130; max_depth = 2; subsample = 0.6; n_estimators = 1900 (S11 Table); when using the selected 150 cancer-related regions as features, learning_rate = 0.011; min_samples_split = 190; min_samples_leaf = 60; max_depth = 3; subsample = 0.6; n_estimators = 2000 (S12 Table).

### Mutational signature analysis

To identify the mutational patterns in cancer-independent and cancer-related regions respectively for each cancer, we first prepared the catalog matrix C with rows as mutation types and columns as different type of regions. In our case, the dimension of C is 96x3, where 96 corresponds to the number of mutation types and 3 corresponds to the three types of regions, which are cancer-independent regions, cancer-related regions, and all the ChromHMM regions in this cancer as the reference. Then we used R package MutationalPatterns [84,85] with default parameters to fit C with the 30 COSMIC mutational signatures and the relative contributions of the 30 mutational signatures can be calculated for the three types of regions in each cancer.

## Supporting information

S1 Fig The framework for the training and testing of the CR model. (TIF)

S2 Fig The toy model illustrates how to train the CR model and identification of cancer-independent and cancer-related regions. The red dots represent the cancer-related regions and the black dots represent the cancer-independent regions. And the black line represents the true model. In practice, we don’t know which dots (i.e. regions/samples) are cancer-related or cancer-independent regions. So we train a CR model using all the samples and get the trained model indicated by the red line. Under the assumption, we know that the trained CR model is not exactly the true model, but it is close to the true model. To identify the cancer-related regions, we take the prediction of the current trained CR model as the background and perform the hypothesis testing for each region (see Online methods for details). We remove the regions with small *p*-value and re-train the CR model using the rest of regions. And then a new CR model (i.e. the blue line) is obtained. This new CR model is closer to the true model. The blue line is treated as the true model and the hypothesis testing is done for each region again based on the prediction of the blue model. At last, the regions with small *p*-value will be taken as cancer-related regions and regions with large *p*-value will be taken as cancer-independent regions.
(TIF)

S3 Fig The scatter plots for the 13 tumor types using the re-trained CR model. Cor: the Pearson correlation. MAE: mean absolute error. MSE: mean squared error. Spearman: Spearman correlation.
(TIF)

S4 Fig The log2(MutationRate+1) distribution across ChromHMM states in breast cancer. All the other cancers have similar wide distributions.
(TIF)

S5 Fig Correlation between predicted and measured mutation rates across ChromHMM states in breast cancer. All the other cancers have similar high correlations.
(TIF)

S6 Fig Cancer-independent regions clustered using the contextual weights of the motifs. For each of the 13 cancer types, the identified cancer-independent regions were clustered into 10 clusters using the Manhattan distance between the feature contextual weight vectors as the similarity metric. Each row is a motif, each column a cluster, and each entry is the average of a motif’s contextual weights in all the regions in a cluster. The clusters were further clustered into 10 groups.
(TIF)

S7 Fig The fold change of chromHMM state in the 9 groups from group B to group J. The color key represents the fold change between the percentage of one state in one group and the percentage of the state in the whole dataset.
(TIF)

S8 Fig Heatmap of normalized mutation signature contributions in 3 different regions across 13 cancers. The color key represents the normalized contribution values.
(TIF)

S1 Table The tumor types and donor size analyzed in this study. (XLSX)

S2 Table The ChromHMM state similarity between cancer and corresponding normal cell lines. Four cancer-normal cell comparisons are shown in terms of length percentage of similar states ("length.similar"). To be more specific, TssA and TssAFlnk are deemed as similar; TxFlnk, Tx and TxWk are similar; ZNF/Rpts, Het, ReprPC, ReprPCWk and Quies are similar.
(XLSX)

S3 Table The summary statistics for 13 tumor types in this study. "Correlation in Train set": the average of Pearson correlations of CR model on the training dataset across 10-fold cross validation. "Correlation in Test set": the average of Pearson correlations of CR model on the testing dataset across 10-fold cross validation. "Merge data to train CR": whether or not to use this tumor type data to train a unified CR model. "Correlation in final model": the Pearson correlation between true value and the prediction from the final CR model applied to all regions in a tumor. "number of regions after removing overlapped regions": the number of regions after removing the overlapped regions with the 5 merged datasets. "Correlation with no overlapped regions": the Pearson correlation between the true value and the prediction from the unified CR model applied to regions without overlaps in a tumor.
(XLSX)

S4 Table The motif ID and the corresponding proteins used in this study. The number of binding sites in each chromosome is also listed. "TotalNum" represents the total number of binding sites in the whole genome.
(XLSX)

S5 Table The 10-fold cross validation results. "chr in test": the chromosome ID used as the testing dataset. "MSE_test": mean squared error in the testing dataset. "Cor_test": the Pearson correlation in the testing dataset. "MSE_train": the mean squared error in the training dataset. "Cor_train": the Pearson correlation in the training dataset.
(XLSX)

S6 Table The chromHMM dataset used in this study and the number of cancer-related regions for each cancer. (XLSX)

S7 Table Training and testing results using merged motif as features. (The annotation of column names can be found in Fig 2B legend).
(XLSX)

S8 Table The important features in each group. "GroupA_beta": the coefficient of feature in group A. 0 represents that the feature is not important in the group. "GroupA1_mean": the average of mutation rate in the regions with the corresponding motif binding sites in group A.
(XLSX)

S9 Table The enriched pathways for the cancer-related regions for 13 tumor types. (XLSX)

S10 Table The motifs significantly disrupted by the somatic mutations. For example, UM_3582.2_3.88_0.56_57_known. TEAD2 is significantly disrupted in three datasets: Lymph-CLL, Kidney-RCC, Ovary-AdenoCA.
(XLSX)

S11 Table The classification performance using all the cancer-related regions as features. (XLSX)

S12 Table The classification performance using selected the 150 most important regions. (XLSX)

S13 Table The accession number for the histone modification used in this study to perform the ChromHMM segmentation. (XLSX)
