# Supplementary figures and images for: Predicting regional somatic mutation rates using DNA motifs
Source: PLoS Comput Biol. 2023 Oct 2;19(10):e1011536. doi: 10.1371/journal.pcbi.1011536 (PMC10569533; doi:10.1371/journal.pcbi.1011536)

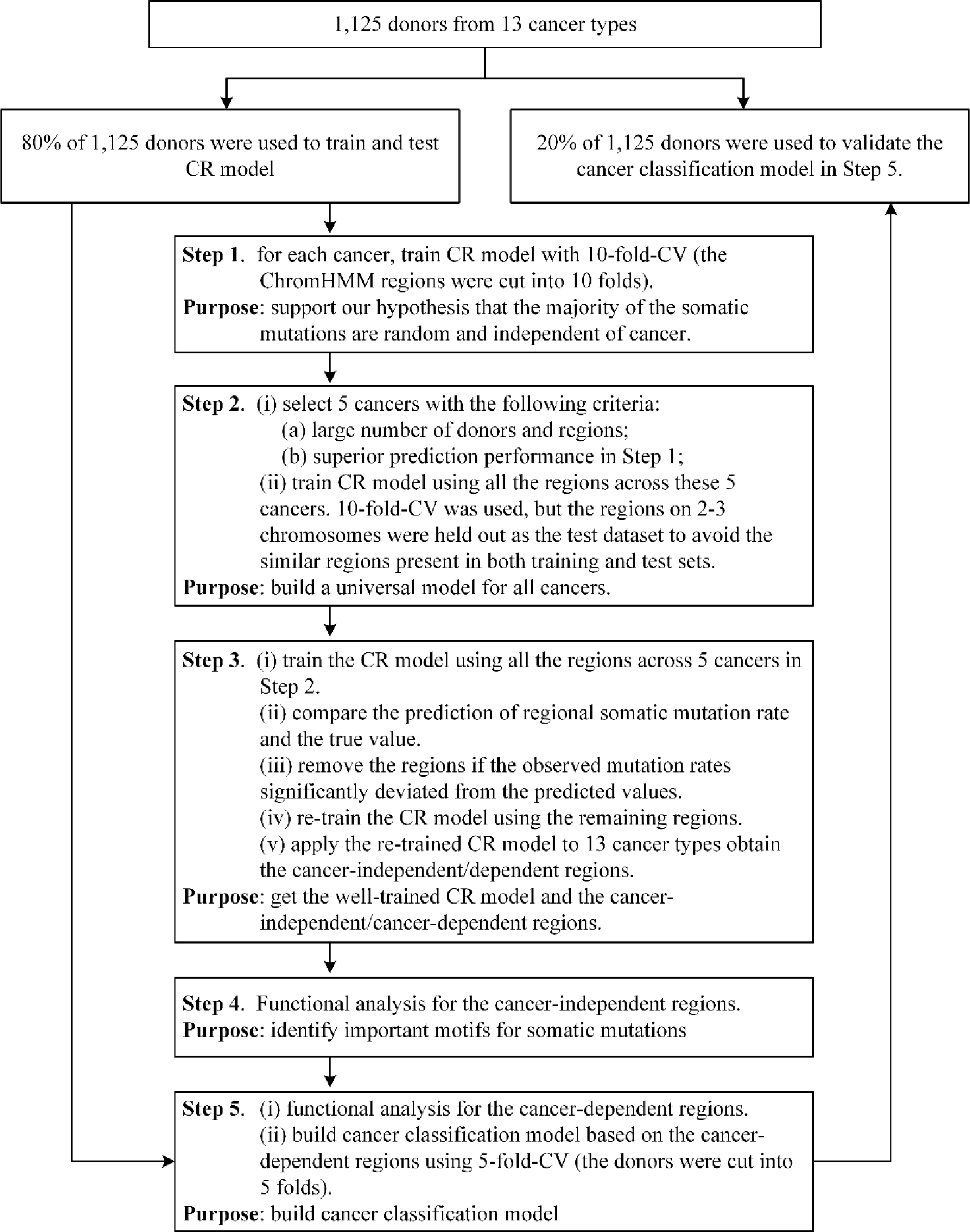

Supplement: S1 Fig — (TIF) [file pcbi.1011536.s001.tif]

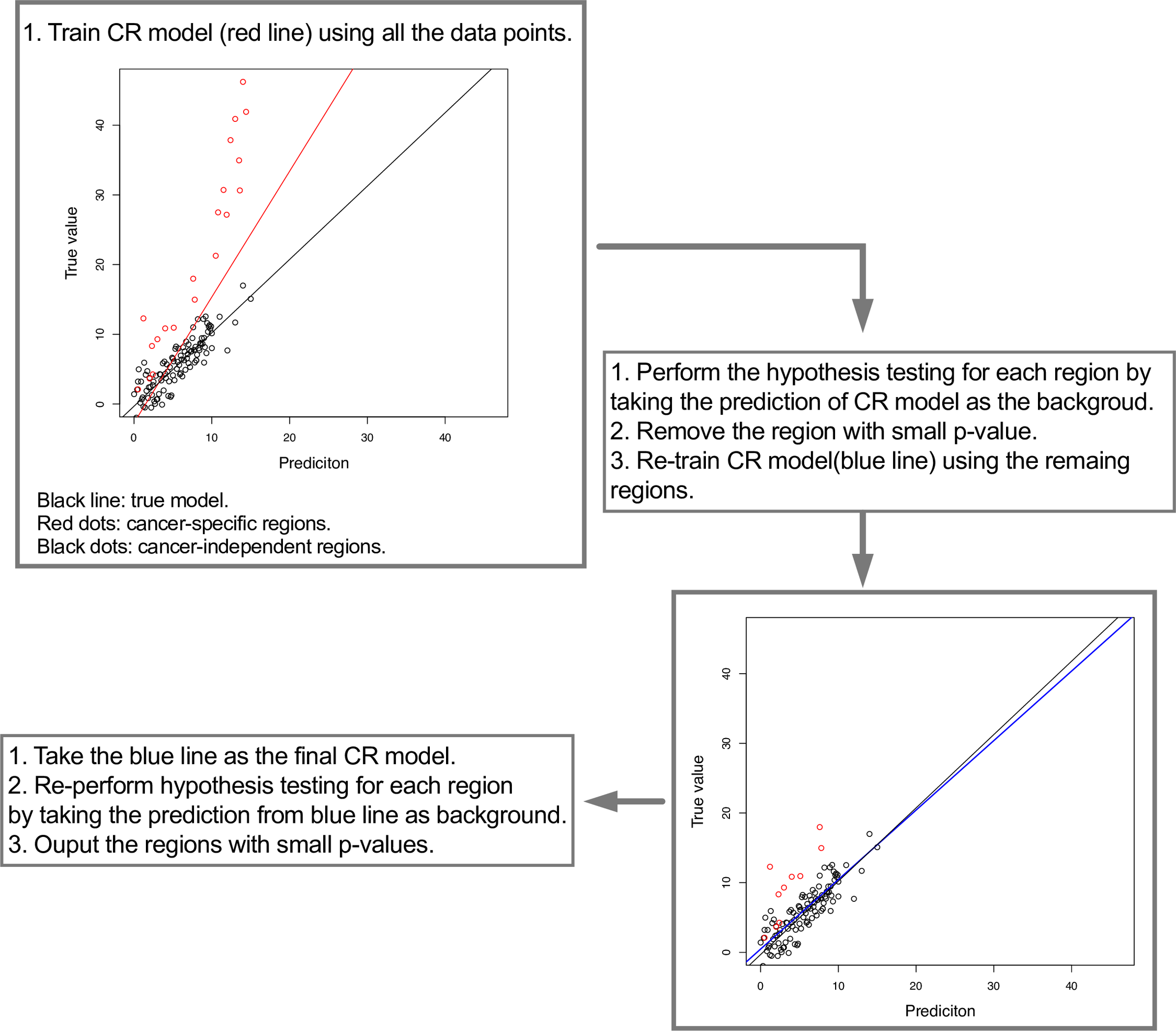

Supplement: S2 Fig — The red dots represent the cancer-related regions and the black dots represent the cancer-independent regions. And the black line represents the true model. In practice, we don’t know which dots (i.e. regions/samples) are cancer-related or cancer-independent regions. So we train a CR model using all the samples and get the trained model indicated by the red line. Under the assumption, we know that the trained CR model is not exactly the true model, but it is close to the true model. To identify the cancer-related regions, we take the prediction of the current trained CR model as the background and perform the hypothesis testing for each region (see Online methods for details). We remove the regions with small p-value and re-train the CR model using the rest of regions. And then a new CR model (i.e. the blue line) is obtained. This new CR model is closer to the true model. The blue line is treated as the true model and the hypothesis testing is done for each region again based on the prediction of the blue model. At last, the regions with small p-value will be taken as cancer-related regions and regions with large p-value will be taken as cancer-independent regions. (TIF) [file pcbi.1011536.s002.tif]

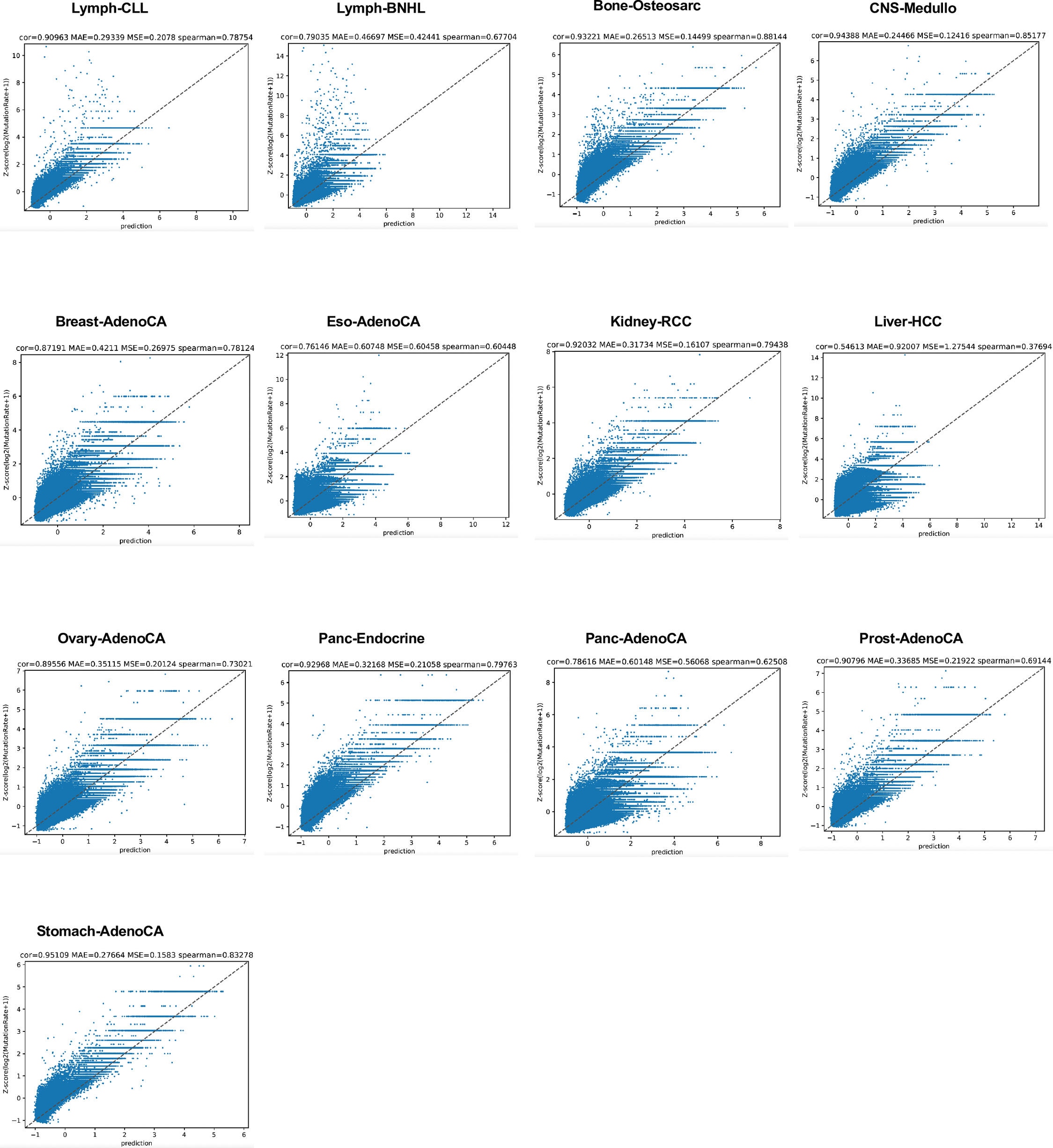

Supplement: S3 Fig — Cor: the Pearson correlation. MAE: mean absolute error. MSE: mean squared error. Spearman: Spearman correlation. (TIF) [file pcbi.1011536.s003.tif]

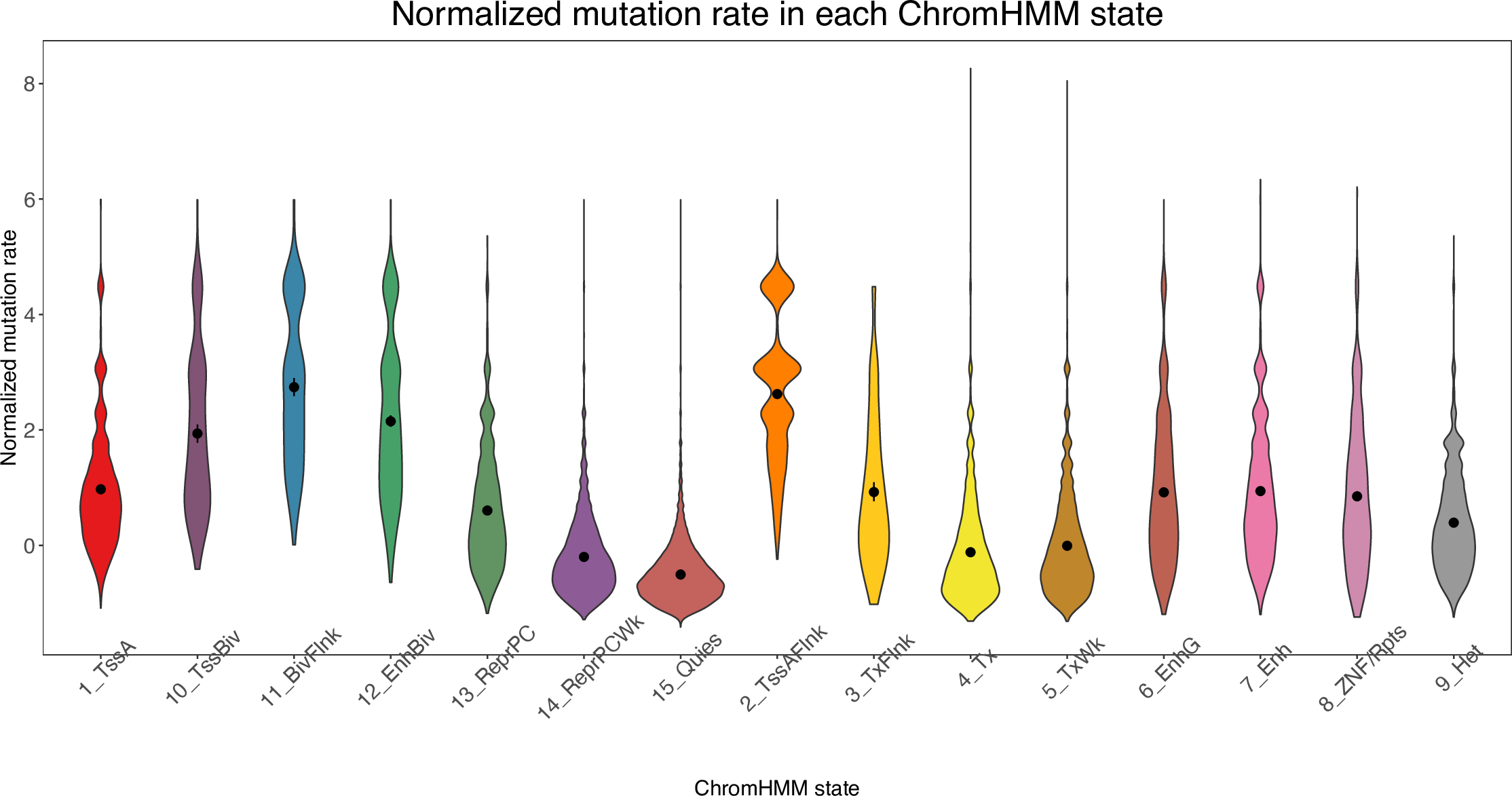

Supplement: S4 Fig — All the other cancers have similar wide distributions. (TIF) [file pcbi.1011536.s004.tif]

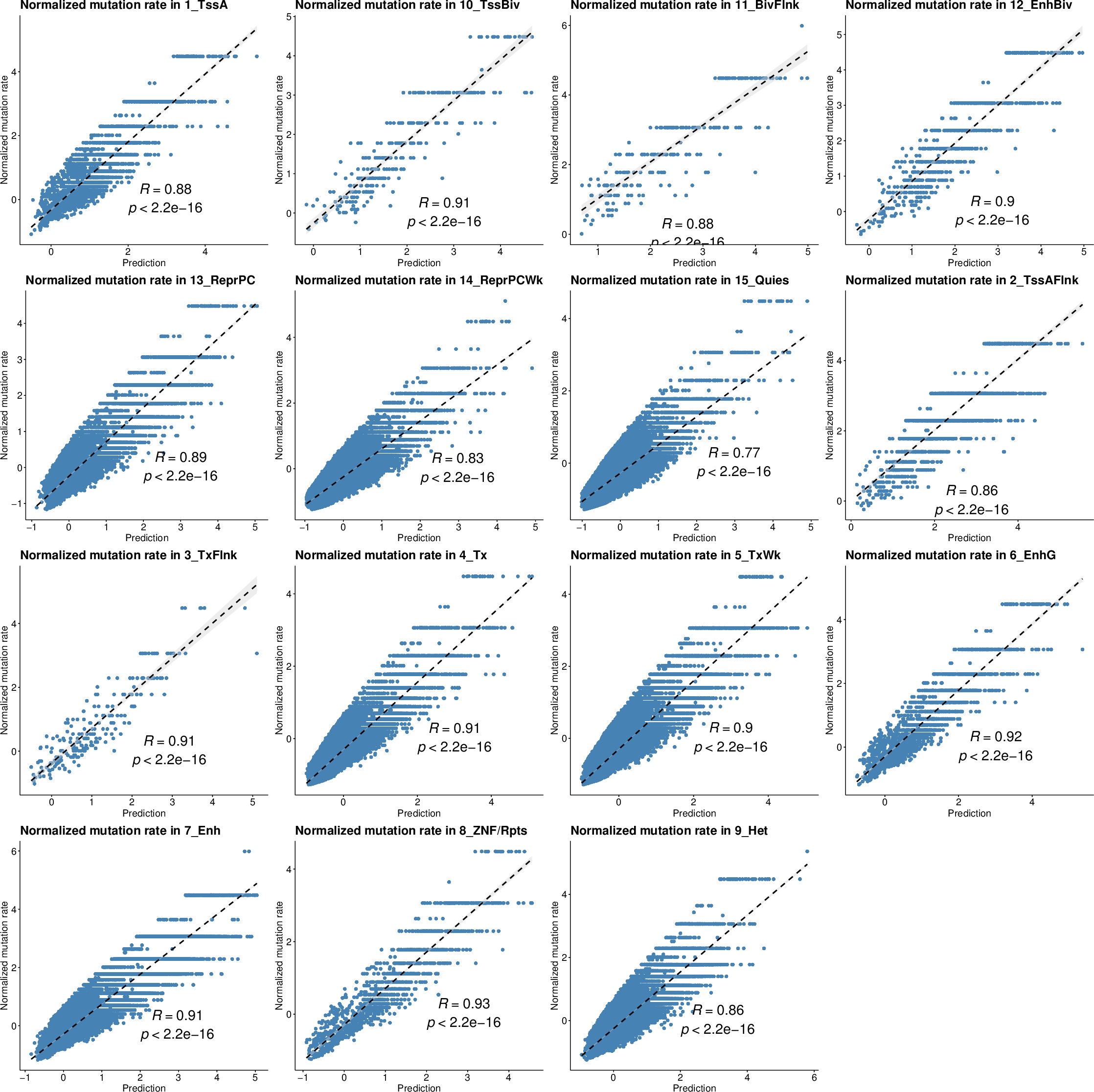

Supplement: S5 Fig — All the other cancers have similar high correlations. (TIF) [file pcbi.1011536.s005.tif]

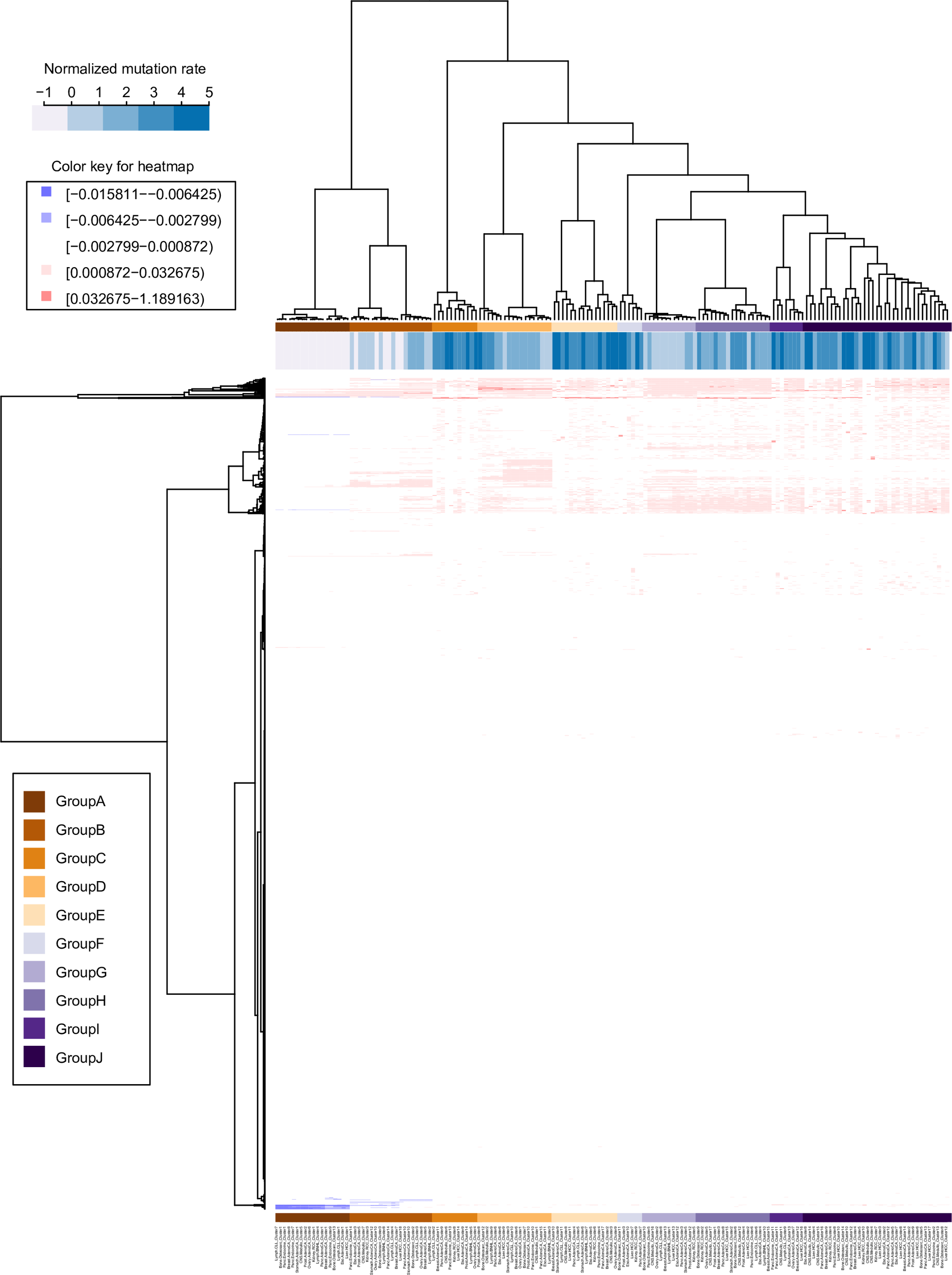

Supplement: S6 Fig — For each of the 13 cancer types, the identified cancer-independent regions were clustered into 10 clusters using the Manhattan distance between the feature contextual weight vectors as the similarity metric. Each row is a motif, each column a cluster, and each entry is the average of a motif’s contextual weights in all the regions in a cluster. The clusters were further clustered into 10 groups. (TIF) [file pcbi.1011536.s006.tif]

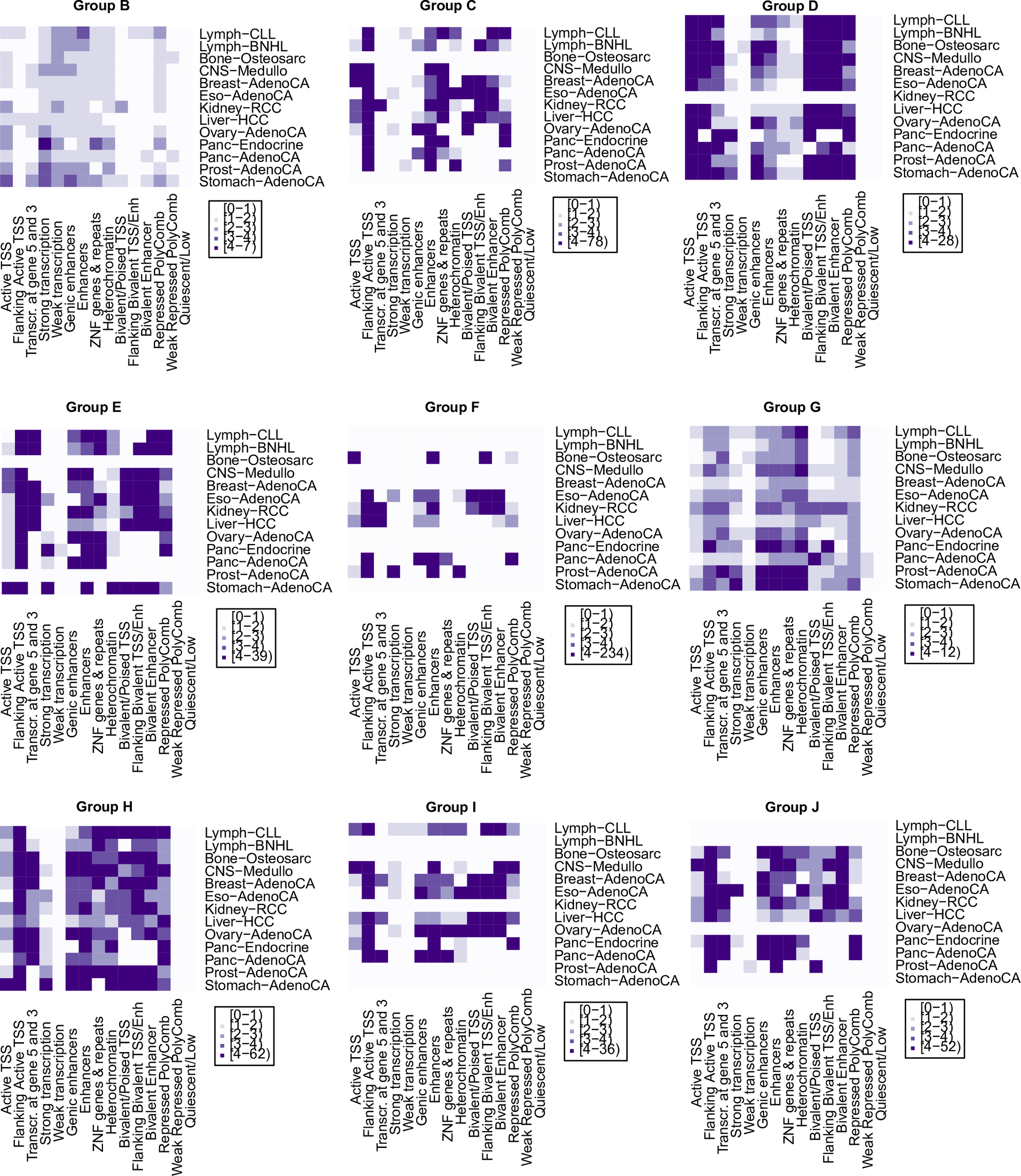

Supplement: S7 Fig — The color key represents the fold change between the percentage of one state in one group and the percentage of the state in the whole dataset. (TIF) [file pcbi.1011536.s007.tif]

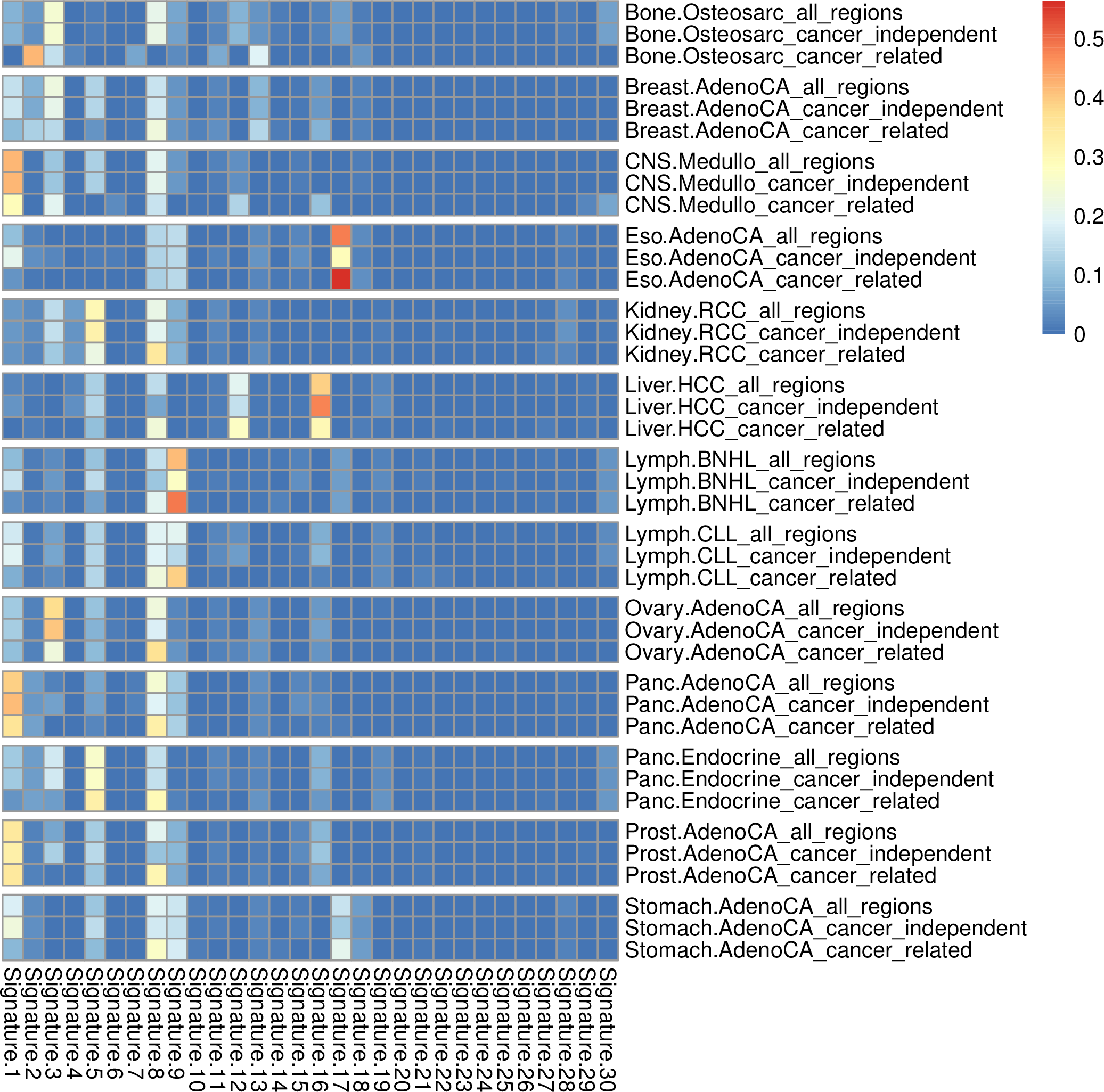

Supplement: S8 Fig — The color key represents the normalized contribution values. (TIF) [file pcbi.1011536.s008.tif]
